# Detection of Soft Tissue Sarcoma Recurrence: Feasibility of Ultrafast 3D Gradient-Echo Sequence in Addition to Conventional Contrast-Enhanced MRI to Provide Early-Phase Postcontrast Information

**DOI:** 10.5334/jbsr.2602

**Published:** 2022-05-27

**Authors:** Hye Min Son, Hye Jin Yoo, Sung hwan Hong, Ja young Choi, Hee dong Chae

**Affiliations:** 1 Yeungnam University medical center, KR; 2 Seoul National University Hospital, KR

**Keywords:** sarcoma, tumor recurrence, multiparametric MRI, contrast media, surveillance imaging

## Abstract

**Objectives:**

Dynamic contrast-enhanced magnetic resonance imaging (DCE-MRI) has been investigated to better detect recurrent tumors of malignant soft tissue sarcoma (STS), however, DCE-MRI is time-consuming and not available at all medical centers. This study aims to evaluate the feasibility of dual-phase postcontrast MRI sequences (early 3D spoiled gradient-echo [GRE] and delayed fast spin-echo [FSE] T1WI) for the differentiation of recurrent tumor from nonneoplastic lesions.

**Materials and methods:**

A total of 297 patients under postoperative surveillance for malignant STS were included in this retrospective study and divided into three subgroups, as follows: group A, recurrent tumors (n = 82); group B, pseudomasses (n = 55); and group C, postoperative inflammation (n = 160). All MRI examinations included dual-phase post-contrast sequences. The contrast-to-noise ratio (CNR) and the signal-intensity ratio (SIR) were used to evaluate the degree of contrast enhancement in target lesions. ROC curve analysis was performed to assess the diagnostic performance for recurrent tumor.

**Results:**

In the early phase, all mean CNR and SIR values were significantly higher in group A (all, *p* < 0.05). However, the difference of the CNR between early and delayed post-contrast MRI showed a significantly lesser increase in group A than in the other groups when muscle was used as the reference tissue (*p* = 0.026). A comparison of ROC curves showed that dual-phase MRI had significantly better diagnostic performance than conventional postcontrast MRI.

**Conclusion:**

The addition of an early postcontrast 3D GRE to conventional FSE-T1WI is useful to detect recurrent tumors by providing additional information on early enhancement.

## Introduction

The survival rate of patients with soft tissue sarcoma (STS) has steadily improved, however, tumor recurrence still significantly worsens the long-term prognosis [1]. Magnetic resonance imaging (MRI) is the primary imaging modality for local surveillance after STS. However, postsurgical changes around the treated tumor on conventional MRI sequences can mimic tumor recurrence [2,3]. Recently, there have been significant advances in MRI techniques to differentiate recurrence from postoperative inflammation and fibrosis [4]. Among them, dynamic contrast-enhanced (DCE)-MRI can increase the accuracy of recurrence detection up to 97% [1,2,5,6,7,8,9,10], because recurrent tumors usually enhance earlier and more rapidly during the first pass of contrast [1,11]. However, DCE-MRI might not be available in all facilities because of the cost-effectiveness and low reproducibility [2]. Furthermore, postprocessing methods and pharmacokinetic analyses such as Emax/1 (maximal relative enhancement at the first minute) are time-consuming [6,7,12]. A large cohort study is needed to validate other simple and easily available MRI sequences to show early enhancement within the first one or two minutes.

At our institution, dual-phase postcontrast MRI that combines early 3D spoiled gradient-echo (GRE) and delayed fast spin-echo (FSE) T1WI sequences are routinely performed. Thus, the purpose of our study was to evaluate the feasibility of dual-phase postcontrast MRI to distinguish recurrent tumors from postoperative inflammation in clinical practice.

## Materials and Methods

### Patient selection

**Figure 1:**
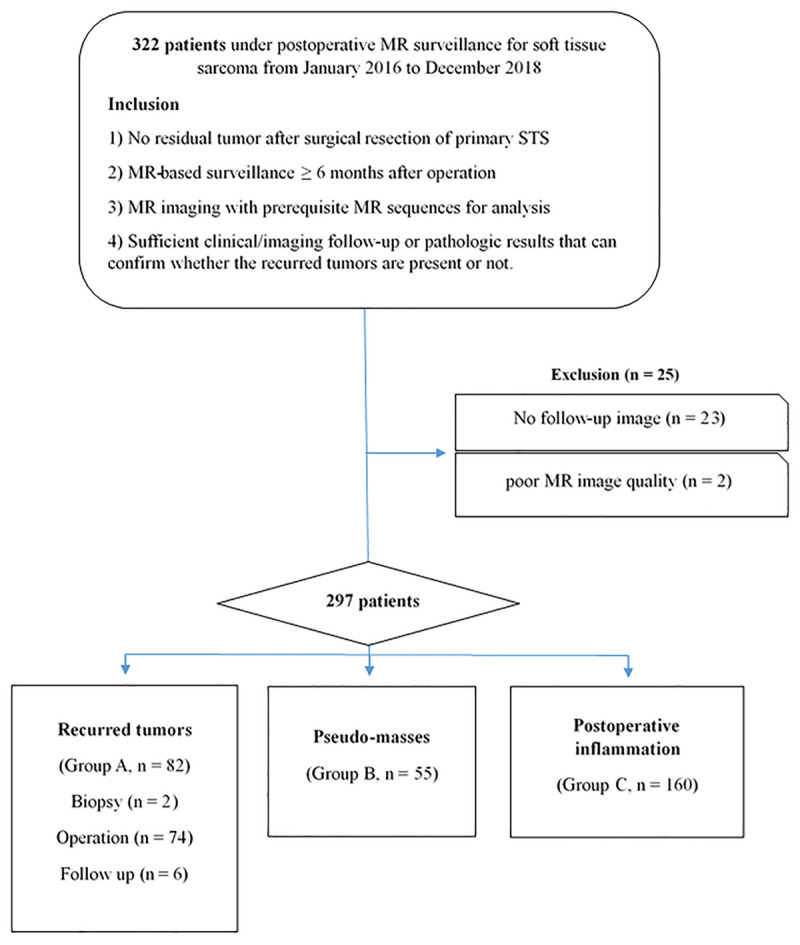
Flowchart showing the inclusion criteria, exclusion criteria, and number of patients in the three subgroups.

This retrospective study was approved by our institutional review board; the requirement for informed consent was waived. Between January 2016 and December 2018, patients who underwent postoperative MRI surveillance for malignant STS at our institution were reviewed independently by two radiologists (with 1 year and 10 years of musculoskeletal MRI experience) for the presence of tumor recurrence. Among the initial 322 consecutive patients, a total of 297 patients who met the following inclusion criteria were included: (a) negative margins on histopathology after surgical resection of the primary STS; (b) MRI-based surveillance for more than six months after the operation; (c) MRI with prerequisite sequences (described in the next section); and (d) sufficient clinical/imaging follow-up or pathological results to confirm recurrent tumors (***Figure 1***).

### Patient subgroups

All included patients were divided into three subgroups: group A = recurrent tumors (n = 82); group B = pseudomasses (n = 55); and group C = postoperative inflammation (n = 160). Recurrent tumors were confirmed by histopathology following a biopsy (n = 2)/operation (n = 74) or proved by an obvious increase in size on follow-up MRI (n = 6). Pseudomasses were defined as nodular or mass-like, T2-hyperintense soft tissue lesions with enhancement in the surgical bed that were suspected as recurrent tumors but ultimately proven to be nontumorous on follow-up MRI at least six months later or by biopsy. Postoperative inflammation was defined as an ill-defined, T2-hyperintense soft tissue lesion without a discrete enhanced nodular lesion in the surgical bed and without evidence of tumor recurrence over the whole follow-up duration.

### MRI technique

All MRI examinations were performed with 3.0-T MRI scanners (Siemens, Philips, and GE Healthcare), as follows: FSE T2WI with modified DIXON (mDIXON), FSE T1WI, 3D spoiled GRE (SPGR) T1WI before and after IV injection of contrast material (within 40~50 sec as late arterial phase; early phase) (Dotarem [gadoterate meglumine], 0.1 mmol per kilogram of body weight) with mDIXON and subtraction imaging, and finally coronal and axial postcontrast FSE T1WI (axial scan after coronal image acquisition to put an approximately five-minute time interval after the contrast material injection; delay phase axial conventional postcontrast FSE T1WI) (Supplement 1).

### Image analysis

Only one MRI scan per patient with the following criteria was selected for image review: (a) with suspected recurrent tumor, the last MRI just before biopsy or surgery; and (b) without tumor recurrence, the last MRI scan with an additional follow-up MRI taken after six months. A circular region of interest (ROI) was drawn on the most prominently enhanced area as large as possible. If multiple lesions were simultaneously detected on the same MRI, the measurement was performed in the largest enhancing lesion. In addition, two circular ROIs were placed in the subcutaneous fat (SQ) layer and muscle on the selected MRI image as reference tissue.

### Enhancement parameters

Contrast-to-noise ratio (CNR) and signal-intensity ratio (SIR) [13,14,15] were calculated to evaluate the degree of enhancement, as follows: CNR = (mean value of the lesion of interest ROI – mean value of the background tissue ROI)/standard deviation (SD) of the background tissue ROI); SIR = mean value of the lesion of interest ROI/mean value of the background tissue ROI. The following abbreviations were used: early (E) and delayed (D) phase; reference background tissue of muscle (m) or SQ (f); early and delayed phase CNR based on the background muscle layer (mCNR(E) and mCNR(D), respectively); early and delayed phase SIR with SQ as the background tissue (fSIR(E) and fSIR(D), respectively). In terms of enhancement pattern, CNR(D-E) = CNR(D) – CNR(E) with each background tissue (fCNR(D-E) for SQ; mCNR(D-E) for muscle).

### Statistical analysis

Descriptive statistics, including demographic data and histopathological types, are reported. The values of all enhancement parameters were compared among subgroups by an independent sample t-test in two ways: (1) recurrence (group A) vs nonneoplastic lesions (groups B+C); and (2) recurrence (group A) vs pseudomass (group B). Temporal changes in the mean values of parameters were analyzed using repeated measures analysis of variance (ANOVA) with the subgroup and scan time (early and delayed phases) as factors. Receiver operating characteristic (ROC) curves of the enhancement parameters were compared between conventional postcontrast FSE-T1WI and dual-phase MRI to estimate their diagnostic performance in distinguishing recurrent tumors from nonneoplastic lesions. A *p* value £ 0.05 was considered indicative of a statistically significant difference.

## Results

### Patient population

A total of 297 MRI scans were obtained from variable body parts (203 lower limbs, 61 upper limbs, and 33 trunks as tumor sites) of 297 patients (age range, 18–94 years; mean age ± SD, 54.1 + 17.4; male:female ratio = 152:145). Approximately 89% of all patients were included in the most common four histopathology groups based on the World Health Organization (WHO) classification of soft tissue tumors (undifferentiated/unclassified sarcoma, adipocytic tumors, tumors of uncertain differentiation, and (myo)fibroblastic tumors) (***Table 1***).

**Table 1:** Demographics of patients and histopathologic types of tumors.


		TOTAL	GROUP A^a^	GROUP B	GROUP C

Total number		297	82	55	160

Sex (M:F)		152:145	43:39	29:26	80:80

Mean age (years) (range)		54.13 ± 17.4 (18–94)	61.46 ± 17 (19–94)	50.64 ± 16.97 (21–82)	51.58 ± 16.7 (18–86)

Tumor sites (number) (lower limbs:upper limbs:trunk)		203:61:33	45:26:11	44:7:4	114:28:18

Distribution of MR vendors Siemens:Philips:GE		141:140:16	38:40:4	27:25:3	76:75:9

**Histopathologic type**	Adipocytic tumor	68^d^	14	16	38

	(myo)fibroblastic tumor	61^e^	14	11	36

	Skeletal muscle tumor	7^f^	4	2	1

	Smooth muscle tumor	12^g^	0	4	8

	Vascular tumor	3^h^	0	2	1

	Chondroosseous tumor	5^i^	1	0	3

	MPNST^c^	13	8	1	4

	Tumors of uncertain differentiation	58^j^	13	11	34

	Undifferentiated Sarcoma	70	28	7	35


a: Group A = recurrent tumor, group B = pseudomasses, group C = postoperative inflammation. c: MPNST = Malignant peripheral nerve sheath tumor. d~j: specific histopathology (number of cases). d: dedifferentiated (10), myxoid (53), and pleomorphic (5) liposarcoma. e: fibrosarcoma (5), myxofibrosarcoma (46), low-grade fibromyxoid sarcoma (7), malignant hemangiopericytoma (3). f: rhabdomyosarcoma (RMS) (1), embryonal (1), alveolar (2), pleomorphic (2), spindle cell/sclerosing (1) RMS. g: leiomyosarcoma (8). h: epithelioid hemangioendothelioma (1) angiosarcoma (2). i: extraskeletal (4) and mesenchymal (1) chondrosarcoma. j: synovial sarcoma (29), epithelioid sarcoma (6), alveolar soft part sarcoma (4), clear cell sarcoma (6), extraseketal myxoid chondrsarcoma (6), malignant mesenchymoma (2), Extra skeletal Ewing sarcoma (5).

### Analysis of enhancement parameters and pattern

**Figure 2:**
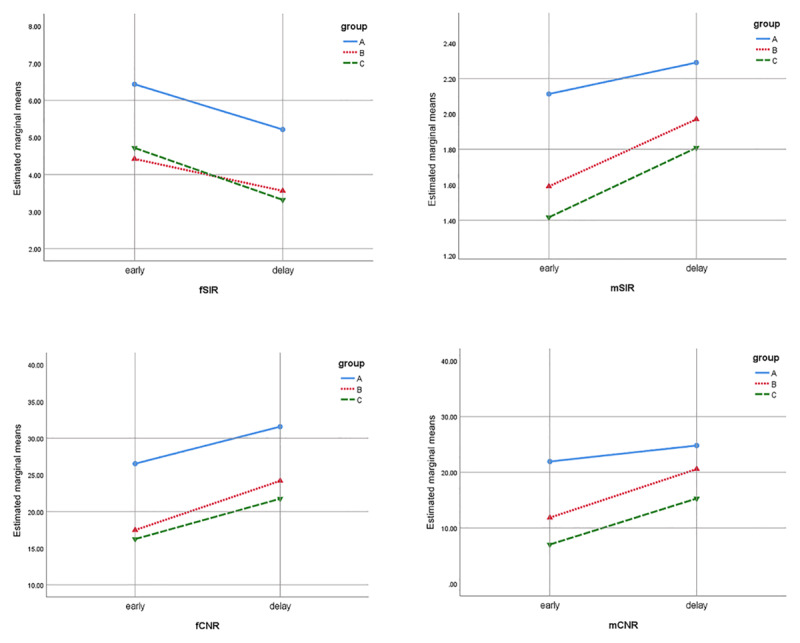
Repeated measures ANOVA output profile plots showed estimates of the marginal means of the SIR and CNR in three subgroups at two time points (early and delayed phases). In group A, only the mCNR slope shows significantly lesser increase than in other groups (d). Group A = recurrent tumor, group B = pseudomass, group C = postoperative inflammation; f = subcutaneous fat as the reference tissue; SIR = signal-intensity ratio; m = skeletal muscle as the reference tissue; CNR = contrast-to-noise ratio.

The mean CNR values were higher than the mean SIR values (range: CNR, 11.85–31.58; SIR, 1.97–6.43). The mCNR(E) showed the largest difference in the mean value between recurrent tumors and nonneoplastic lesions. Recurrent tumors showed significantly higher CNR and SIR values than nonneoplastic lesions. In addition, all CNR and SIR values except for the mCNR(D) were significantly higher in recurrent tumors than in pseudomasses (*p* < 0.05). Recurrent tumors also showed a more gradual enhancement slope from the early to delayed phase (ΔCNR = 2.874) than did pseudomasses and postoperative inflammation (ΔCNR = 8.73 and 9.50, respectively) but only when muscle was used as the reference tissue (mCNR). The difference in the SIR over the scan time interval was much smaller than that in the CNR (range: CNR, 2.874–8.729; SIR, 0.178–1.401) and showed a downward slope for the fSIR, opposite of all other values (***Table 2*** and ***Figure 2***).

**Table 2:** Mean values of calculated parameters among subgroups.


	GROUP A^a^	GROUP B	GROUP C	*p* VALUE (A VS B)	*p* VALUE(A VS B+C)

fCNR(E)^b^	26.52 ± 15.16	17.47 ± 11.46	16.24 ± 12.02	**0.000**	**0.000**

fCNR(D)^c^	31.58 ± 21.46	24.18 ± 14.02	21.75 ± 18.49	**0.026**	**0.000**

fCNR(D-E)^d^	5.05 ± 21.41	6.72 ± 15.17	5.51 ± 16.18	0.620	0.769

mCNR(E)^e^	21.94 ± 11.78	11.85 ± 12.46	7.12 ± 7.53	**0.000**	**0.000**

mCNR(D)	24.81 ± 15.89	20.58 ± 13.31	17.02 ± 16.12	0.107	**0.000**

mCNR(D-E)	2.87 ± 15.37	8.73 ± 14.08	9.50 ± 15.58	**0.026**	**0.006**

fSIR(E)^f^	6.43 ± 4.45	4.42 ± 2.39	4.72 ± 3.52	**0.003**	**0.001**

fSIR(D)	5.21 ± 5.05	3.56 ± 1.57	3.31 ± 1.73	**0.020**	**0.000**

mSIR (E)	2.11 ± 0.54	1.59 ± 0.48	1.42 ± 0.49	**0.000**	**0.000**

mSIR (D)	2.29 ± 0.71	1.97 ± 0.51	1.86 ± 0.89	**0.005**	**0.000**


Mean value ± standard deviation. a: Group A = recurrent tumor, group B = pseudomasses, group C = postoperative inflammation. b: f = subcutaneous fat as a reference tissue; E = early phase; CNR = Contrast-to-noise ratio. c: D = delay phase. d: CNR (D-E) = CNR on FSE-T1WI – CNR on 3D-GRE. e: m = skeletal muscle as a reference tissue. f: SIR = signal-intensity ratio.

### Comparison of ROC curves

**Figure 3:**
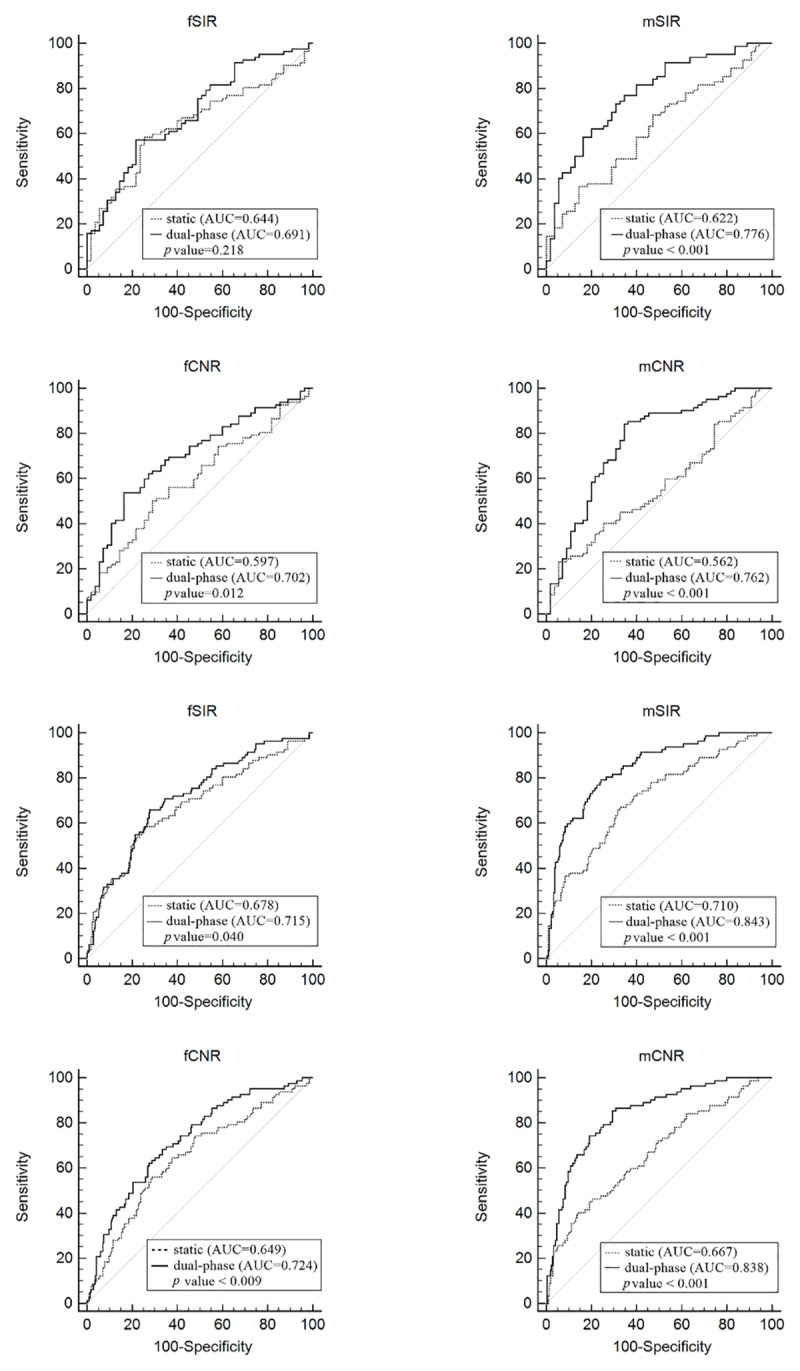
ROC Curves comparing the diagnostic value of conventional postcontrast FSE T1WI (dashed lines) and dual-phase postcontrast MRI (solid lines) in differentiating between recurrence and pseudomasses (upper 4 panels) and between recurrence and nonneoplastic lesions (lower 4 panels). Dual-phase postcontrast MRI showed significantly better performance in all parameters, except for the fSIR.

**Figure 4:**
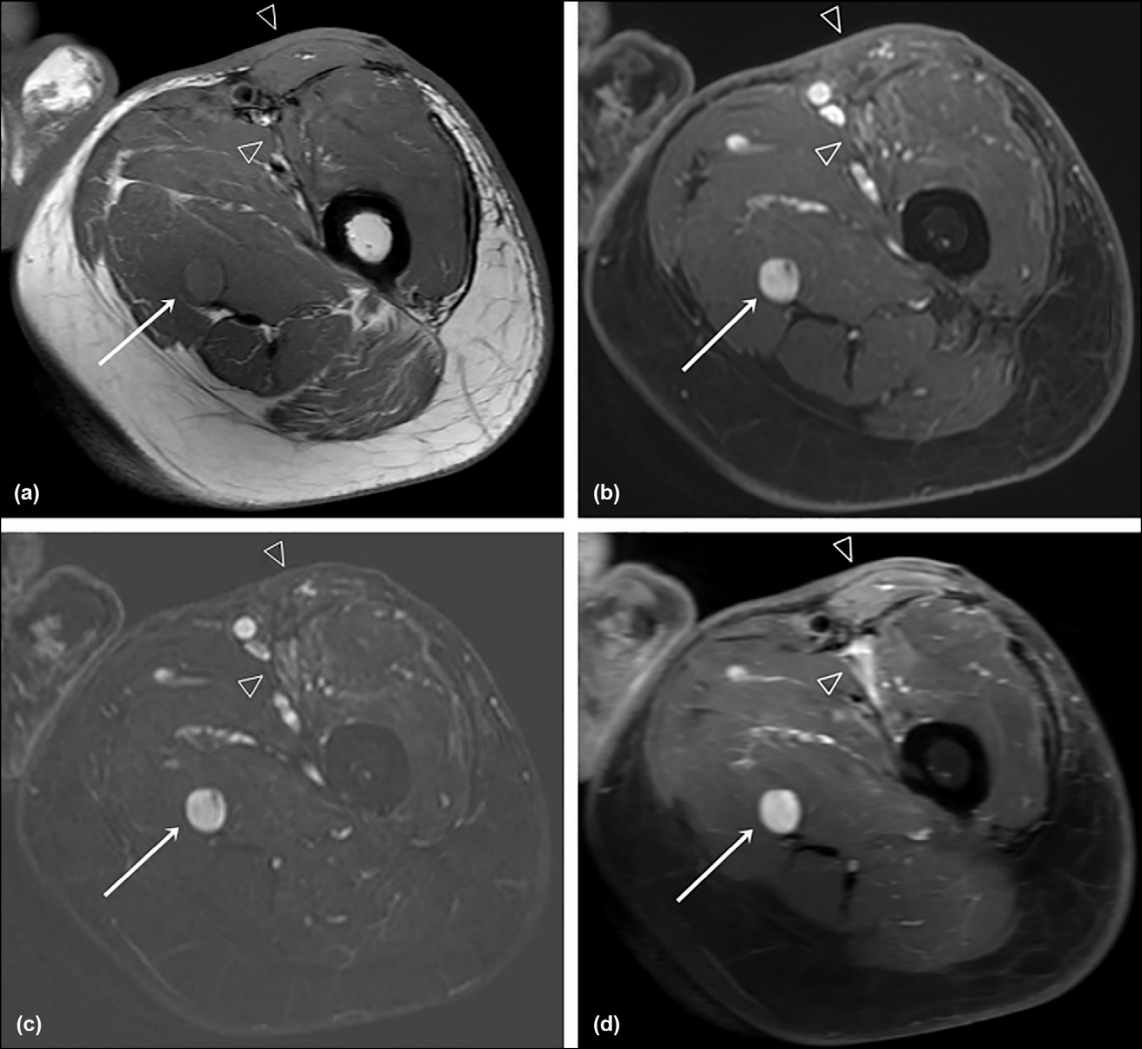
A 59-year-old male with recurrent myxofibrosarcoma after wide excision **(a)** Axial precontrast FSE T1WI, **(b)** axial postcontrast 3D spoiled GRE, **(c)** axial subtraction GRE imaging, and **(d)** axial postcontrast FSE T1WI showed a round, well-defined, strongly enhanced nodule (histologically proven recurrence) in the left proximal thigh, which is well enhanced on both early (b) and delayed (c) phases. In contrast, postoperative inflammation at the anterior aspect of the proximal thigh showed gradual enhancement (arrowheads).

**Figure 5:**
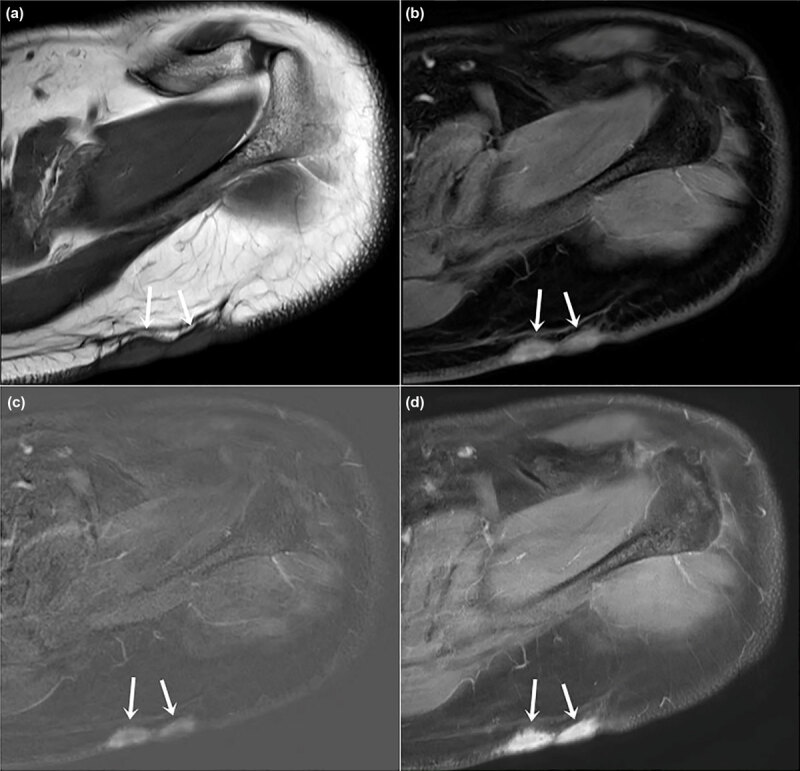
A 24-year-old male with a pseudomass after wide excision for Ewing sarcoma **(a)** Axial precontrast FSE T1WI, **(b)** axial postcontrast 3D spoiled GRE, **(c)** axial subtraction GRE imaging, and **(d)** axial postcontrast FSE T1WI showed two well-defined, ovoid, gradually enhancing nodules at subcutaneous layer of the left posterior shoulder along the proximal margin of excision site, which was confirmed as a pseudomass on a needle biopsy.

Paired ROC curves of conventional FSE-T1WI and dual-phase postcontrast MRI were compared for each parameter in the differentiation of recurrent tumors from nonneoplastic lesions (group A vs groups B+C) or from pseudomasses (group A vs group B) (***Figure 3***). Dual-phase postcontrast MRI showed significantly better diagnostic performance than conventional contrast-enhanced MRI for all parameters except for the fSIR. Also, dual-phase MR with mCNR showed the best diagnostic performance (AUC = 0.838). MRI images of representative target lesions for each of groups A and B are shown in ***Figures 4*** and ***5***.

## Discussion

In this study, recurrent tumors showed significantly higher CNR and SIR values and a more gradual enhancement slope from the early to delayed phase than nonneoplastic lesions, which means that a high postcontrast signal intensity was already reached within the first one to two minutes in recurrent tumors. In addition, considering much lower mean value, smaller temporal change, and downward slope as opposite of all other values, the CNR is considered a more reliable and stable measure than the SIR to represent the degree of contrast enhancement of the target lesion. And the adjacent normal muscle layer may better serve as background tissue than the SQ layer.

This retrospective study has several limitations. First, a heterogeneous group of patients with different histopathological types and various body parts were included. However, in recurrent tumors, areas with the strong enhancements are likely to represent the higher grade of tumor rather than morpho-phenotypic differentiation [16,17], and the four most common histopathological tumor types accounted for 89% of all included tumors. Second, 3.0-T MR scanners from three different vendors were used, each using a manufacturer-specific 3D SPGR sequence, which inevitably causes minor differences in data acquisition and protocol details. Third, the 3D SPGR was performed at 40~50 sec after the injection of contrast media (approximately 2 minutes for the image acquisition), so it is considered late arterial phase since it does not exactly represent the early arterial phase on DCE-MRI, which is usually carried out with 5–30 sec of temporal resolution [4]. Fourth, CNR and SIR to compensate for inconsistent MRI scan parameters are not absolute values. Thus, they cannot be directly compared with the TIC shapes. Recently semi-quantitative metrics were attempted to minimize variation across multi-center and multi-vendor acquisitions. As part of this attempt, CNR and SNR is used as a marker of the quality or detectability of the contrast of interest. For MR imaging, this quantification can be used to allow comparison among variable imaging hardware, protocols and acquisition sequences [18].

In conclusion, the addition of an early postcontrast 3D SPGR is useful to distinguish recurrent tumors from nonneoplastic lesions by providing additional information on temporal change of enhancement.

## Appendix: Supplement 1

### Materials and Methods: MRI techniques

The following parameters were used for these two sequences (3D spoiled GRE vs. FSE T1WI): slice thickness/spacing, 4 mm/2 mm vs. 4~6 mm/2~4 mm; flip angle, 9~12 vs. 90~140 degrees; TR, 4.6~6 vs. 600~670 msec; TE, 1~2.6 vs. 11.5~14.5 msec; acquisition time, 2 minutes vs. 3 minutes 30 sec; and variable fields of view (FOVs) considering the covered body parts.
